# Radar-Based Damage Detection in a Wind Turbine Blade Using Convolutional Neural Networks: A Proof-of-Concept Under Fatigue Loading

**DOI:** 10.3390/s25113337

**Published:** 2025-05-26

**Authors:** Erik Streser, Sercan Alipek, Manuel Rao, Jonas Simon, Jochen Moll, Peter Kraemer, Viktor Krozer

**Affiliations:** 1 Department of Physics, Goethe-University Frankfurt, Max-von-Laue Str. 1, 60438 Frankfurt am Main, Germany; 2 Department of Mechanical Engineering, University of Siegen, Paul-Bonatz-Straße 9-11, 57076 Siegen, Germany

**Keywords:** structural health monitoring, damage detection, wind turbine blade, FMCW radar, millimeter-wave, convolutional neural network

## Abstract

This paper reports a convolutional neural network (CNN)-based damage detection approach for radar-based structural health monitoring of wind turbine blades. Subsequent radar measurements are transformed into an image-type representation for use as CNN input. In contrast to conventional approaches that require compensation for temperature and loading effects, the proposed framework inherently learns all required information during the training phase. Its damage detection performance (i.e., detecting intact vs. damaged condition) is demonstrated using measurements from multiple embedded radar sensors during fatigue testing of a wind turbine blade with a length of 31 m. The achieved F1-score for correct damage classification is between 91% and 100% for both the unloaded and the loaded blade.

## 1. Introduction

Renewable energies such as wind and solar are needed to reduce the greenhouse gas effects caused by the growing global demand for energy and to achieve climate neutrality in the European Union by 2050 [1]. Due to advanced manufacturing technologies, the costs of onshore and offshore wind turbines (WTs) have decreased by 23% and 32%, respectively, since 2015 [2]. This motivates the continuous expansion of sustainable energy infrastructure for affordable and clean energy.

Wind turbine blades (WTBs) represent the main component of a wind turbine. In 2012, 19.4% of all WT accidents were caused by rotor blade damage. The main reason for such damage is material fatigue due to causes such as rotation, heavy rain, lightning strikes, ice accumulation, strong winds, aerodynamic excitation of vibrations, collisions with birds, and manufacturing defects [3]. Typical types of damage include debonding, delamination, cracking, contamination, erosion, corrosion, and splitting [4]. While longer WTBs produce more energy per WT, they also incur these types of damage more frequently [5].

Structural health monitoring (SHM) of WTBs is essential to guaranteeing their safety and reliability, and various sensor systems have been developed for this purpose. In particular, vibration-based methods see frequent use, as they allow for continuous inspection at early stages [6,7]. However, vibration-based methods only provide global damage-related information. Popular local non-destructive testing (NDT) methods include those based on acoustic emissions, thermography, and machine vision as well as on ultrasonic, strain, radiographic, and electromagnetic measurements [8,9].

Several demonstrations of SHM methodologies have been reported in the literature. For example, Samareh-Mousavi et al. [10] performed full fatigue testing of a single artificially-initiated damage instance in the spar cap of a 31 m long WTB using the cyclic bending load. Delamination location and growth were determined by visual inspection, acoustic emission, and thermography. Mielke et al. [11] studied acoustic emission data from a biaxially fatigued WTB with a length of 14.3 m and four embedded wrinkle defects along the spar cap. Correlation between both the energy and frequency and the position of the delaminations was verified. Field studies by Yang et al. [12] and Zhang et al. [13] have shown that cracks can be detected by microphone recordings. The audio signals in these studies were composed of random wind noise and the mechanics of the WT. The first study used the short-time Fourier transform (STFT) in a deep learning (DL) framework for detectability, while the second compared data in the frequency domain for both the intact and damaged structure. In the fatigue test performed by Cai et al. [14], infrared thermography was used to measure local heat dissipation due to vibration loading of a simplified TC11 titanium alloy blade structure. Zabihi et al. [15] used piezoelectric transducers and a laser Doppler vibrometer for vibration-based detection of cracks with different depths. The emitted and received laser pulses were analyzed in terms of their Hilbert spectra. Fiber Bragg grating strain sensors in the WTB roots were applied in the work of both Pacheco et al. [16] and Cai et al. [17]. Pacheco et al. [16] recorded the data of two WTs for 14 months and visualized load scenarios caused by varying environmental and operational conditions (EOCs). Strain measurements have been carried out for tidal energy production as well [18,19].

Electromagnetic methods such as radar-based methods have the advantage of non-contact and low-cost inspection of composite structures [5]. Simon et al. [20] investigated a full-scale fatigue test with 40 FMCW radars in a WTB and evaluated the damage severity by computing damage indicators (DI). In a further study, a trend decomposition algorithm was developed for temperature compensation [21]. The same measurement data are used for this work.

**Figure 1:**
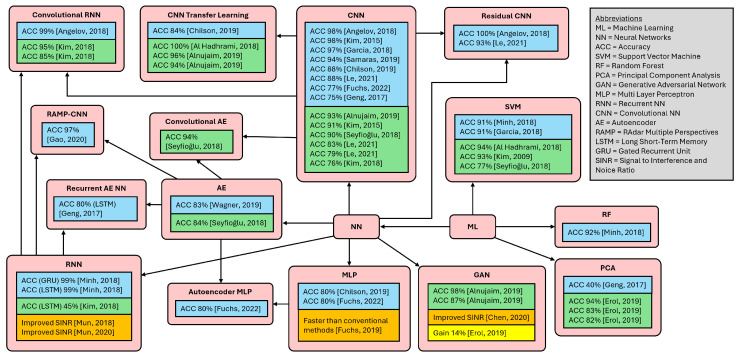
Overview of different ML architectures, with percentage of accuracy in radar applications. Blue boxes indicate object classification, green boxes indicate motion classification, orange boxes indicate data preprocessing, and yellow boxes indicate data augmentation [29,30,31,32,33,34,35,36,37,38,39,40,41,42,43,44,45,46,47,48,49,50].

Many recent data analysis approaches have been based on machine learning (ML). ML involves the creation of an inductive model that learns from a finite amount of data and maps any input as accurately as possible to an estimated output. Depending on the labeled input, a distinction is made between supervised, unsupervised, and semi-supervised ML [22]. Different architectures reported in the literature are compared in Figure 1, which depicts their accuracy for object and motion classification, data preprocessing, and data augmentation. Deep learning (DL) is a subset of ML that uses artificial neural networks with multiple layers to learn distinct representations from data. Due to their high computational demand, graphics processing units (GPUs) are relied on to leverage parallelization for improved efficiency [23]. Convolutional neural network (CNN) architectures are particularly suitable for damage detection in structural health monitoring [24]. CNN models generally include convolution, pooling and fully-connected layers [23].

Various ML architectures have been used for WTB monitoring. Khazaee et al. [25] simulated a vibration-excited WT with blade defects and achieved 50–100% accuracy with a CNN depending on the wind turbulence intensity. Saharan et al. [26] simulated different cracking cases, achieving a peak detection accuracy of 98.8% with their CNN. Li and Gao [27] classified aerodynamic and mass imbalances between damaged and normal conditions using a support vector machine (SVM) and a CNN. They tested different sized convolution kernels, achieving a maximum accuracy of 98.63%. Rizk et al. [28] examined a WTB sample for cracking, erosion and ice accumulation using a 3D-CNN. The accuracy reached 100% for full-band images, which fell by less than 1% even with reduced dimensionality.

The novelty of the present work lies in the successful design, implementation, and application of an ML framework for radar-based WTB monitoring. Our approach leverages the discriminative power of CNNs for damage detection under fatigue loading. In contrast to other approaches, the proposed CNN-based method does not require compensation for environmental effects such as temperature changes or for operational effects such as loading. The experimental validation is based on a 31 m WTB that incurred fatigue damage due to excitation for two months under laboratory conditions at Fraunhofer IWES in Bremerhaven. A network of 40 FMCW radars at 60 GHz were installed in the WTB for continuous data acquisition in order to monitor its structural condition. The validation process showed that the CNN was able to distinguish between intact and damaged structural conditions with high accuracy.

The remainder of this paper is structured as follows: Section 2 describes the experimental setup for the WTB fatigue experiment, including the radar network and the signal processing pipeline; Section 3 presents the damage detection results along with the hyperparameter optimization and accuracy for three different scenarios, including an unloaded blade and a loaded blade; finally, conclusions are drawn in Section 5.

## 2. Materials and Methods

### 2.1. Experimental Setup

**Figure 2:**
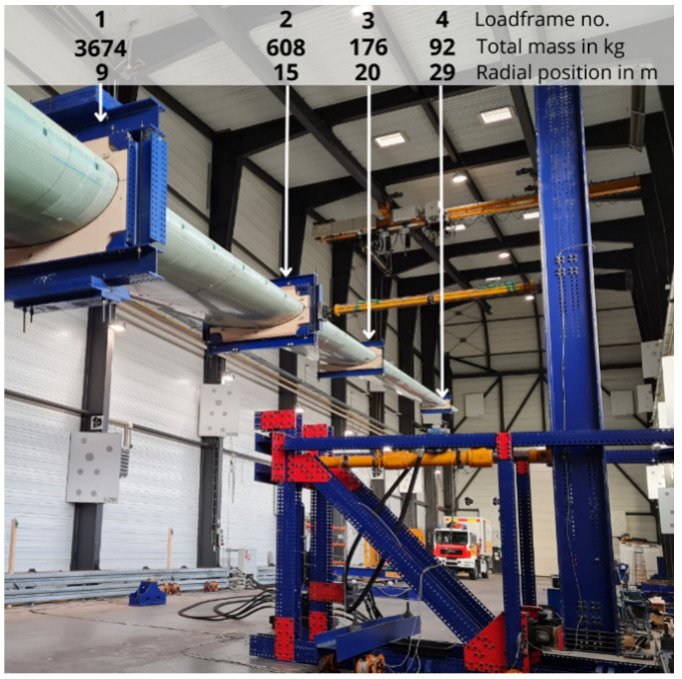
Setup of the fatigue test. The photo shows the horizontally positioned WTB with four attached load frames. The markings on the photo highlight the location of the load frames, with load frame 1 being the closest to the blade root at a distance of 9 m [20].

The explicitly designed and manufactured “IB30 WTB” by Fraunhofer IWES consists of dry non-crimp glass fiber, PET foam, and balsa wood as core materials, along with a PU resin matrix system and PU adhesive material for bonding of the web and shell components. The butterfly manufacturing process with several molding procedures is described in [20]. In thicker regions, up to 128 laminate layers are applied [51]. The WTB was investigated for durability and fatigue using four load frames and a ground-based hydraulic cylinder. This adjustment of the bending moment distribution along the blade simulated real-world wind effects in a controlled manner. Excessive local overloading over time was avoided. Fatigue testing on this 31 m blade was performed according to the IEC 61400-23:2014-04 standard [52] in a 70 m hall in Bremerhaven. After a total of 1,250,000 cycles, the fatigue test was completed and various damage types occurred [20]. A photo of the experimental setup with additional information on the load frames is provided in Figure 2.

**Figure 3:**
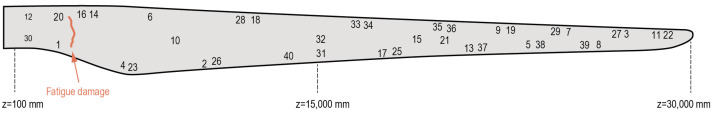
Arrangement of sensor nodes on the WTB. For redundancy purposes, several locations were covered with pairs of sensor nodes, for instance numbers 28 and 18 or numbers 4 and 23, after [20].

The WTB included 40 Infineon FMCW radar sensors operating at 60 GHz installed along the inner surface. All sensors were of type BGT60TR13C; 30 sensors were assembled as retrofit nodes, while 10 were embedded during manufacturing of the blade. Each radar sensor was combined with other measurement components (including an acceleration sensor with three orthogonal axes) into an individual node with an additional microcontroller unit, which managed data acquisition and wireless data transfer. The embedded sensors showed limited performance over several periods during the experiment, and as such were not considered further. The retrofit nodes were glued to the inner surface of the WTB along with a lithium polymer battery and a power supply. Every sensor had four antennas, representing one Tx and three Rx channels. The bandwidth spanned 5.5 GHz with a frequency range from 58 GHz to 63.5 GHz. The modulation slope was 42.9 MHz/μs, resulting in a chirp duration of 0.128 ms. The maximum detection range was 3.5 m for each radar sensor, which was the result of 128 range bins in 2.73 cm steps. It is important to mention that each sensor node placed on one side of the inner blade surface measured the opposite side of the inner blade surface. Figure 3 provides an overview of the sensor arrangement on the rotor blade along with the location of the fatigue damage. Pairs of nodes that appear next to each other in Figure 3 are separated by height, which means that one sensor is located on the lower inner surface of the rotor blade and the other sensor is located on the upper surface.

The sensor nodes were split into four groups based on proximity, with ten nodes per group. Each group communicated with a separate base station through a wireless network connection. The base stations each consisted of a single STM32F767ZI Nucleo board with an AX5043 sub-GHz transceiver (STMicroelectronics, Grenoble, France) mounted on top. Networks were spaced with a 350 kHz frequency offset in order to reduce interference between different base station–group pairs. Only one sensor node per group could communicate with its respective base station at a time.

The rotor blade was horizontally fixated at the blade root, which pointed upwards with the suction side during the complete experiment. A hydraulic cylinder connected to load frame 2 was used to excite the rotor blade perpendicular to the orientation of the WTB at 1.61 Hz, with the load increased over six steps. The frequency of 1.61 Hz was used because it is close to the natural frequency of the rotor blade. The first three load levels, quantified with 102.5%, 106%, and 112%, were set for 400,000 consecutive excitations each. A load level of 100% refers to the condition in which the blade was expected to endure sustained loading over time without significant damage. Early structural changes appeared during the phase with 106% loading, in form of small line cracks on the trailing edge. Critical structural damage was intended after the first three phases; therefore, much shorter phases with increased loads were performed, with 1000 excitations at 117%, 122%, and 127%. The final phase led to a 1.5 m long crack a few meters away from the blade root and in a different location than the earlier line cracks.

Radar measurements were continuously recorded during excitation as well as at regular times when the rotor blade was in an unloaded state, i.e., without excitation. Further environmental and operational parameters were recorded throughout the campaign, as these can have a significant impact on measured signals and possibly mask damage-related information [53].

### 2.2. Data Preprocessing and Data Engineering

Before an ML system can use a dataset to solve a certain task, the data must first be preprocessed. Initially, the dataset was preprocessed to remove data from malfunctioning nodes. Five out of 40 sensor nodes did not send any data, and three others sent only a few samples. The remaining 32 nodes provided between 1,200,000 and 6,800,000 measured frequency ramps over the whole experiment. With a successful measurement holding 120 ramps on average, the number of readouts per node was from 10,000 to 57,000. Additionally, all measurements with anomalies in other environmental and operational conditions were discarded, including node voltage and node temperature. This was particularly the case during WTB excitation.

The raw data including the radar measurements, voltage, temperature, and acceleration were first stored as multiple individual files. For a better access, all measurements were then consolidated into large HDF5 files with one file per parameter. The HDF5 files were grown incrementally by appending one measurement at a time to the tables. The radar sensor had three channels, but not every channel delivered the same amount of data. While many sensors had similar counts across all channels, several had matching numbers between only the first two channels, with the third channel having lower numbers.

**Figure 4:**
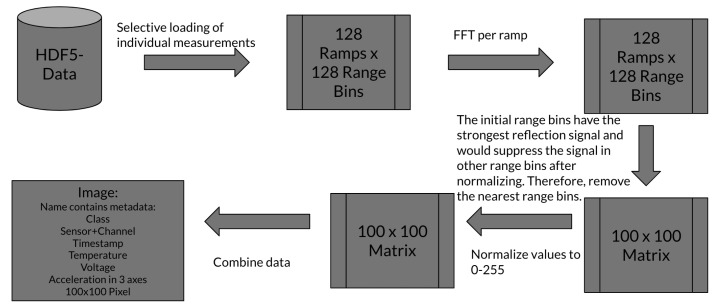
Preprocessing pipeline of the radar data. This flowchart displays all preprocessing steps before the data reached the final input format for the ML model.

A high-level description of the radar data preprocessing is provided in Figure 4. A single ideal measurement was defined by 128 ramps, with 128 range bins per ramp; therefore, a single measurement per channel was defined as a 128 × 128 matrix, with each ramp forming an individual row. Certain measurements did not consist of 128 ramps due to technical reasons. Only measurements that actually held at least 115 ramps were further considered. Then, each ramp was transformed to the distance domain via FFT and application of a Hann window. Out of these measurements, the first 100 ramps were selected with the inner range bins 15 to 115. This selection was stored as a 100 × 100 matrix for each measurement. An unusually high signal strength in the first few range bins was likely caused by mutual antenna coupling between transmitting and receiving antenna. Truncation of the first few range bins helped the model to focus on the relevant discriminative features without superimposed coupling effects from the first range bins or losing too much of the reflection data, which were still contained in the closer remaining range bins. For the next step, each matrix was normalized to hold values between 0 and 255. In the last step, the normalized 100 × 100 matrix was stored as a Numpy array which had all metadata listed in the file name.

**Figure 5:**
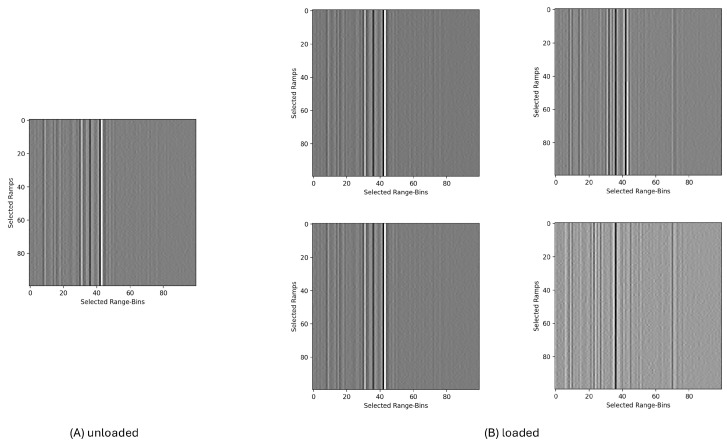
Final input format of five single radar measurements (from sensor 14, channel 1) for the ML model. Each plot represents a radargram as a 100 × 100 matrix that represents a stack of 100 ramps with 100 range bins per ramp. Each pixel contains values between 0 and 255. (**A**) shows a typical measurement during the idle or unloaded state, while (**B**) shows four different measurements of the same sensor and channel under loading.

An example of five single measurements is plotted in Figure 5. This example was taken from the first channel of node 14 when the blade was in an unloaded or loaded state. Each pixel represents the radar data for one range bin of one ramp; the darker the pixel, the stronger the reflected signal.

### 2.3. CNN-Based Data Processing Pipeline

Inspired by the CNN architecture for radar image interpretation presented by Garcıa et al. [46], a new architecture for WTB health classification is proposed in this work. In the related work, a CNN successfully classified occupied parking spaces with an accuracy of 97% by introducing spatial coordinates as features in the fully-connected layer, compared to 95% accuracy without them. These improvements suggest the importance of additional parameters such as spatial information in radar data interpretation. In addition, because this is a relatively simple and proven architecture, it makes sense to adapt it to the problem of damage detection in WTBs. For now, additional parameters are not used in the architecture; however, in a real-world implementation parameters such as temperature, wind speed, rain, and ice can be added. The benefits to the model’s performance of adding those parameters must also be further analyzed.

**Figure 6:**
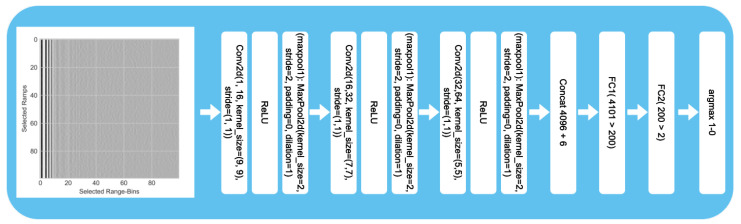
Architecture of the ML model.

The architecture of the CNN shown in Figure 6 is designed to perform binary classification of the prepared radar images as either *intact* or *damaged*. The software was written in Python using Pytorch, with CUDA for the network design and parallel computation with a GPU. In the beginning, three convolutional layers are used to interpret the 100 × 100 image. Afterwards, features collected by the convolutional layer can be extended by additional parameters and introduced as input to two fully connected neural layers. However, no additional parameters were added in our ML experiments. Each layer consisted of a convolutional 2D layer followed by a rectified linear unit (ReLU) activation layer and finalized by a max pooling layer.

The convolutional layer in the first block has a kernel size of 9 × 9 and creates 16 output channels. With a stride of 1 and no padding, the kernels create 16 feature maps of size 92 × 92 as output based on the sliding window’s movement. The output feature map values result from a linear combination of 9 × 9 values on the part of the input image. The model extracts spatial features using a width of 9 range bins. The received radar signal can be noisy depending on the radar device and application. Taking 9 ramps for each range bin can decrease the influence of noise on the results. With no padding, the image values at the corners of the image are used less frequently, resulting in reduced impact on the feature maps. However, this also reduces the size of the output feature maps.

Each of the 16 kernels has a size of 9 × 9, meaning that the convolutional 2D layer has 1296 weights to train. The 16 feature maps of size 92 × 92 contain 135,424 values. The features are passed through the ReLU layer, which sets all negative values to 0, effectively suppressing them in the subsequent layers of the network. To reduce the size of the feature maps, they are decreased by the max pooling layer. With a stride of two, the maximum activation function skips every other value. Using a stride size of two allows the features to be passed to the resulting feature map with size 46 × 46. As before, we use a padding of 1 and dilation of 1.

The 16 feature maps of size 46 × 46 then form the input to the second convolutional block. Each of the 16 feature maps is used to create a new single feature map. In total, 32 feature maps make up the output of the convolutional layer. With a kernel size of 7 × 7 and 16 feature maps as input, the total number of weights is 25,088. The padding and stride remain the same as in the first layer. This results in 32 feature maps of size 40 × 40 and 51,200 values. To keep the computational cost and size reasonable, after a ReLU layer, the feature maps are decreased in size by another max pooling layer. This layer is designed in the same way as the previous one. This results in 32 feature maps of size 20 × 20.

In the third and last convolutional block, the kernel size is reduced to 5 × 5, while the number of output feature maps is increased to 64. Padding is not used, and the output size of the feature maps is 16 × 16. As before, the output feature maps are passed through a ReLU layer and reduced by a max pooling layer, this time to a size of 8 × 8. In the third layer, 51,200 weights must be trained.

The 64 feature maps are then resolved into one array of size 4096. Other features could be added to this feature array of the convolutional part of the model, such as the weather (rain, snow), outside temperature, and node’s temperature or battery voltage. The feature array that forms the input of the two layers of the fully connected neural network consists of 4096 values. Each feature is linked to one node of the first layer, and each input node of the first layer is connected to each of the 200 output nodes. The 200 output features of the first fully connected layer are then reduced to two outputs in the second fully connected layer. By selecting the maximum output of the last fully connected layer, both the initial image and any potential additional features are classified. The possible labels are *intact* and *damaged*.

The total number of trainable parameters in this architecture is around 900,000. Of these, the convolutional layers account for approximately 78,000 values. Despite being a short CNN, the architecture has a comparatively high computational cost, resulting in longer training durations. However, the choice of architecture was driven by factors such as simplicity and transparency, allowing for a proof-of-concept of the proposition that neural networks in general are able to detect damage-related changes in a rotor blade during loaded and idle phases by using its radar reflection. Such a proof does not require the most efficient state-of-the-art architectures. Future work may consider optimization of our approach for more efficient, faster, and more robust execution by using techniques such as depthwise separable convolutions (as in MobileNetV1 [54]), squeeze and excitation blocks [55], residual connections (as in ResNets [56]), or even entirely different neural networks such as transformers [57], which are based on attention modules rather than convolution layers.

### 2.4. ML Methodology

The proposed architecture was trained on unique datasets for different case studies (loaded and unloaded WTB). While the training process largely remained consistent, it was designed to be adaptable. The number of iterations was adjusted slightly based on the specific case study. This mainly depended on the amount of data available from the experiment.

We evaluated model performance using the F1-score and accuracy, along with the confusion matrix. A grid search was used to find the optimal hyperparameters. This means that the hyperparameters were changed step-wise in order to find the optimal combination. The adjustable hyperparameters were the optimizer function, learning rate, batch size, and gradient clipping. For hyperparameters with a range, the values we used were set by the user, and were not always linear. Each combination of hyperparameters was used in a training run. For each run, we used the available experimental data with a split of 60% training data, 20% validation data, and 20% test data. The data were shuffled before splitting. Shuffling was necessary in this case because the data were created based on HDF5 files, which stored all the data in chronological order, and because both the *damaged* and *intact* classes are based on time and had a roughly even distribution.

An initial grid search with a wide array of hyperparameters was performed in each ML scenario. An algorithm was implemented to speed up the grid search to check whether the model improved during training. When the model remained within ±0.5% validation accuracy for seven training iterations, the hyperparameter configuration was assumed to be near to or at its peak accuracy. The resulting configuration of hyperparameters was then fixed and the run was stopped. The best three variations of hyperparameters were chosen based on the grid search. These were then further validated by running each set of hyperparameters three times with different seeds for a random weight initialization.

## 3. Results

This section describes the results for three different scenarios. The first experiment created a baseline for the model’s performance, while also analyzing its damage detection capability in the unloaded state for sensors near the WTB’s structural critical crack. The second experiment considered a different subset of sensors, as the number of measurements was sufficiently large for both the unloaded and loaded cases. Finally, the third experiment used the same sensors as the second dataset while the WTB was excited.

### 3.1. Damage Detection (Unloaded)

The first experiment aimed to establish a baseline for the model’s performance and validate the data processing pipeline. The experimental approach included data from the early beginning of the experiment as well as data closer to the end. Only sensors near the structural critical crack of the WTB were used. The data contained radar measurements of the WTB in pristine condition, labeled as *intact*, along with measurements near the end of the experiment when the blade was damaged, labeled as *damaged*. Both classes were equally represented. Data were only selected from time periods when the blade was unloaded. The *intact* data class consisted of measurements between 17 December 2021–18 December 2021, while the *damaged* data class included measurements from 6 February 2022–7 February 2022. A summary of the data, selected sensors, and channels is shown in Table 1.

In addition to the continuous deterioration of the WTB’s structural condition the temperature of the hall changed slightly [21]. During the first period around Christmas and New Year, the hall temperature was about 18 °C. The temperature was decreased because of vacation time. For the second period, when damage was present, the hall temperature was slightly higher at around 20 °C.

**Figure 7:**
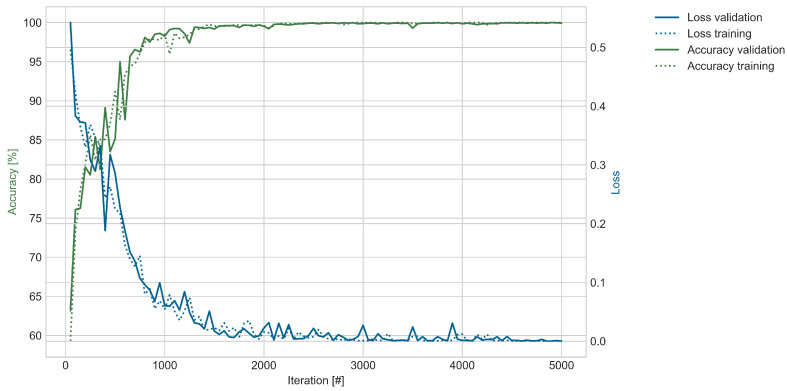
This figure shows the model’s performance for the unloaded dataset described in Table 1 during training of one validation run. The training is based on the following hyperparameters: Optimizer—Adam; learning rate—0.00005; batch size—128; gradient clipping—10.

Figure 7 shows the loss and accuracy of the training and validation datasets during training for one hyperparameter configuration. The figure is representative for both datasets when the WTB was unloaded. The accuracy improved quickly during the first few hundred iterations and reached a plateau. The trends of the training and validation accuracy are similar, with the training accuracy mostly above the validation accuracy.

Each iteration was trained with the batch size number of classified images. According to the 60% split, around 8780 images were used for training. Dividing the total amount of training data by the batch size of 128 leads to the number of iterations. This means that after 69 iterations the total training dataset was used once to train the model. In 5000 iterations, the training dataset was used about 72 times to train the model. Based on the grid search, the best three configurations of hyperparameters were selected for validation. The results of the validation run are shown in Table 2 and are discussed in the next paragraph.

In this case, the model’s exceptional performance is evident, achieving an accuracy of 99.97% and an F1-score of 99.98% as an average of the validation runs for the best configuration. The model can nearly perfectly distinguish between the *intact* and *damaged* WTB conditions. The three best-performing configurations achieved accuracy and F1-scores higher than 99.6% with minor standard deviations, indicating robust results for the ML architecture. The ML experiment was repeated with sensors away from the crack in order to further validate the model’s ability to distinguish between *intact* and *damaged* conditions, as described in the following section.

### 3.2. Damage Detection (Unloaded) Verification

The ML experiment described in Section 3.1 was repeated with different sensors and considering a separate dataset. Table 3 lists the parameters for the second experiment. The amount of data was comparable to the first experiment, the distribution between the *intact* and *damaged* classes remained roughly even, and the WTB remained unloaded.

Again, the best three performing hyperparameter configurations were validated by rerunning them with three different seeds. The results are displayed in Table 4.

Similar to the first experiment, the model achieved high accuracy and F1-scores, detecting smaller cracks and overall structural deterioration effects despite the sensors not being near the critical structural crack. The results show that the model was capable of distinguishing between the *intact* and *damaged* states for the selected sensors.

### 3.3. Damage Detection (During Fatigue Testing)

Using the same sensors as described in the previous section, the architecture was then trained with measurements taken while the WTB was loaded. The number of measurements was significantly decreased, with just over 900 measurements. Table 5 summarizes the dataset. Using the same procedure as before, the best-performing hyperparameter configurations were searched and validated, as summarized in Table 6.

**Figure 8:**
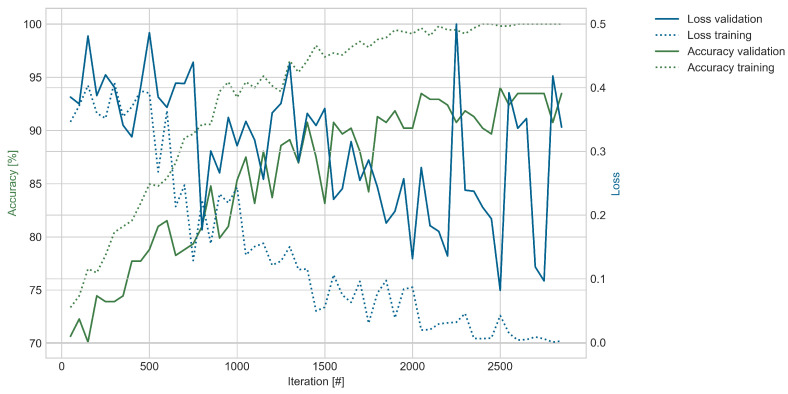
Model performance for the dataset described in Table 5 during fatigue loading. The figure shows the training based on the following hyperparameters: Optimizer—Adam; learning rate—0.00005; batch size—128; gradient clipping—5.

Figure 8 shows the progress of the training in the same way as Figure 7. However, due to the decreased amount of available data, the number of training iterations was decreased, which reduces the risk of overfitting. As for the unloaded dataset, the training loss and accuracy approach their respective theoretical optimum levels of 0 and 100%; however, the validation performance is lower. The validation accuracy follows a similar trend as the training accuracy but with an increasing gap, reaching a maximum value of around 94%. With much more variation, the validation loss remains higher than for the unloaded dataset and varies between 0.4 and 0.1 in the last iterations.

The results for the dataset during fatigue testing of the WTB remain high, with the highest average accuracy being 94.02% and the highest F1-score being 93.08%. This is a decrease compared to the experiments when the WTB was unloaded. In addition, the standard deviations were higher.

Differentiating the classes during loading is expected to be more complex, leading to lower performance. As discussed before, the WTB deforms under loading, effectively creating different radar responses. Therefore, the images for the same sensor and channel are slightly different. Furthermore, the WTB heats up, which influences the radar measurements. This was shown by Simon et al. [58], who demonstrated the temperature dependency of radar measurements. Additionally, the amount of available data to train the model was lower. Despite all these circumstances, the model still achieves a high classification accuracy and F1-score, indicating its ability to learn characteristic features and overcome operational variations despite the manipulated WTB. The ability of the proposed networks to learn the relevant features without requiring further data preparation and feature extraction by human operators makes this architecture easy to use.

However, the effects of real-world conditions on the proposed architecture need to be further analyzed. The radar sensors can detect weather conditions such as rain and snow, which introduce irregular deviations into the measurements. Considering that fatigue testing already decreases the model’s performance, incorporating weather conditions would most likely have the same effect, although these adverse effects might be reduced with more training data.

Although the WTB’s temperature increased during excitation due to friction effects, the hall temperature difference of around 2% for the unloaded experiments remained consistent. This could influence the radar signal, and may be a feature of the radar images created to train the architecture. However, the temperature difference is relatively small, and is even less relevant during mechanical loading due to the heating of the WTB itself.

The concept of adding further parameters from other sensors as input to the CNN part of the architecture should be researched and implemented in future architectures. The benefits are laid out in the overview of condition monitoring of WTBs provided by [59]. Combining different methods of measurement could allow for a more comprehensive understanding of the WTB’s state. Including additional features could also improve the performance of the network, as shown by Garcia et al. [46].

## 4. Discussion

It is important to evaluate the proposed work using different factors. First, a fatigue test of this scale is very costly, as a single wind turbine rotor blade can only be used once due to its being excited until reaching a complete failure state. Second, the sensor system used in our experiments did not have inbuilt edge devices capable of real-time AI applications. Therefore, a second revised implementation of this experimental setup to include edge applications could be a worthwhile progression of this work. For a future edge device application, optimization steps and related tests towards a more robust and lightweight model design are crucial. Thus, additional tests regarding the inference speed, power consumption, or memory usage of the model are omitted for simplicity and because the current network was chosen only for a proof-of-concept. A horizontal study to investigate different and more sophisticated architectures in terms of their size, inference speed, training speed, and robustness is imaginable for future works. Despite these issues, by performing successful damage classification of a wind turbine blade under fatigue loading, the present work represents a novel approach from the SHM viewpoint.

## 5. Conclusions

This paper has introduced a CNN-based damage detection approach for wind turbine blades (WTBs) that is able to identify damage in the presence of changing environmental and operational conditions, in particular, temperature and load variations during fatigue testing of a 31 m long WTB. FMCW radar measurements from multiple radar sensors operating from 58 to 63.5 GHz were used as input to a convolutional neural network (CNN). This approach achieved good classification accuracy (between 91% and 100%) in all three considered application cases.

## Figures and Tables

**Table 1:** Parameters of the dataset consisting of sensors near the critical structural crack of the unloaded WTB.

Parameter	Value
Amount of data	14,634
State of blade	Unloaded
%-Class: damaged	57.67%
%-Class: intact	42.33%
Used Sensor (Channel)	1 (1, 2, 3), 14 (1, 2, 3), 16 (1, 2, 3)

**Table 2:** Performance results of the model trained on the dataset described in Figure 7. The WTB was unloaded, and the sensors near the critical structural crack have been selected. Each of the three best-performing hyperparameter combinations was trained three times with different initial starting parameters, then the results were averaged for each hyperparameter combination.

Optimizer	Learning-Rate	Batch-Size	Gradient-Clipping	AVG. Accuracy [%]	STD. Accuracy [%]	AVG. F1-Score [%]	STD. F1-Score [%]
ADAM	0.00005	128	10	99.97	0.02	99.98	0.02
ADAM	0.00005	128	5	99.89	0.05	99.90	0.04
ADAM	0.00005	256	10	99.63	0.50	99.66	0.46

**Table 3:** The dataset consisting of sensors across the WTB when the WTB was unloaded.

Parameter	Value
Amount of data	12,637
State of blade	Unloaded
%-Class: damaged	52.54%
%-Class: intact	47.46%
Used Sensor (Channel)	12 (1, 2, 3), 26 (1), 28 (1, 2, 3)

**Table 4:** This table shows the performance results with the WTB unloaded and sensors across the WTB selected. Each of the three best-performing hyperparameter combinations was trained three times with different initial starting parameters, then the results were averaged for each hyperparameter combination.

Optimizer	Learning-Rate	Batch-Size	Gradient-Clipping	AVG. Accuracy [%]	STD. Accuracy [%]	AVG. F1-Score [%]	STD. F1-Score [%]
ADAM	0.00005	256	10	99.97	0.03	99.97	0.03
ADAM	0.00005	256	None	99.97	0.03	99.97	0.03
ADAM	0.00005	128	5	99.94	0.05	99.95	0.05

**Table 5:** Dataset consisting of sensors across the WTB during fatigue testing.

Parameter	Value
Amount of data	920
State of blade	Loaded
%-Class: damaged	44.89%
%-Class: intact	55.11%
Used Sensor (Channel)	12 (1, 2, 3), 26 (1), 28 (1, 2, 3)

**Table 6:** The best performing hyperparameter configurations of the CNN model for the loaded case for sensors 12, 26, and 28. Each run was performed three times, and the average results and standard deviations are shown.

Optimizer	Learning-Rate	Batch-Size	Gradient-Clipping	AVG. Accuracy [%]	STD. Accuracy [%]	AVG. F1-Score [%]	STD. F1-Score [%]
ADAM	0.00005	128	5	94.02	2.17	93.08	2.56
SGD	0.008	128	5	93.48	4.31	92.52	5.01
ADAM	0.0001	64	10	92.93	1.09	91.91	1.71

## Data Availability

The datasets presented in this article are not readily available because of their size and storage space requirements. Requests to access the datasets or preprocessed parts should be directed to jochen.moll@uni-siegen.de.

## Abbreviations

The following abbreviations are used in this manuscript:

AVG	Average
CNN	Convolutional neural network
EOC	Environmental and operational conditions
FMCW	Frequency-modulated continuous wave
GPU	Graphics processing unit
ML	Machine learning
NDT	Non-destructive testing
SHM	Structural health monitoring
STD	Standard deviation
SVM	Support vector machine
WT	Wind turbine
WTB	Wind turbine blade
